# Pericardial cyst on the left heart border

**DOI:** 10.1007/s12471-019-1301-y

**Published:** 2019-06-13

**Authors:** T. H. Pinxterhuis, A. P. van der Weerdt, C. A. da Fonseca

**Affiliations:** Department of Cardiology, Medical Centre Leeuwarden, Leeuwarden, The Netherlands

**Fig. 1:**
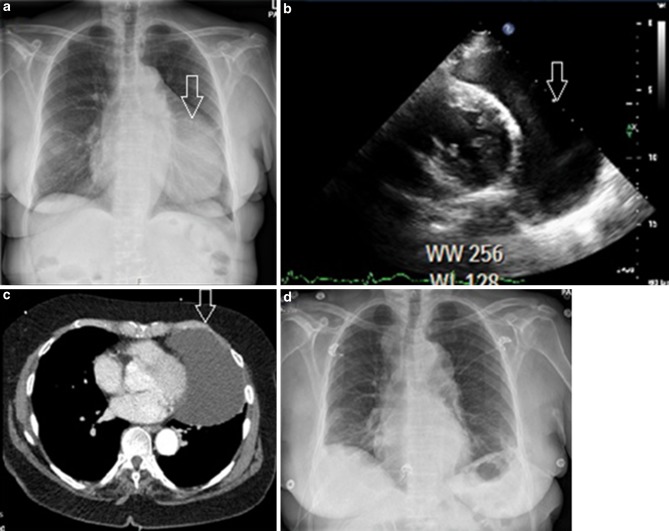
**a** Chest radiography showing an enlarged cardiac silhouette, **b** TTE showing the presence of an echo-lucent space adjacent to the left ventricle, **c** CT heart image showing a lobulated, low-signal-intensity, cystic mass within the pericardium to the left of the left heart border, **d** Chest radiography showing a normal heart contour after surgical removal of the pericardial cyst (*CT* computed tomography, *TTE* transthoracic echocardiography)

A 65-year-old female was admitted with progressive exertional dyspnoea and chest pain. Chest radiography revealed an enlarged cardiac silhouette suspicious for a large pericardial effusion (Fig. 1a). Transthoracic echocardiogram was normal, apart from a large echo-lucent space strictly limited to the anterolateral border of the left ventricle (Fig. 1b). Computed tomography scan showed a large cystic mass at the left heart border (17 × 7 cm, see Fig. 1c). Patient underwent video-assisted thoracic surgery in which the entire cyst was removed. During surgery the diagnosis pericardial cyst was confirmed. Chest radiography afterwards showed a normal heart contour (Fig. 1d).

Pericardial cysts are mostly diagnosed in women around 50 years [1]. Ninety percent of the patients are asymptomatic. If symptoms occur, chest pain is most common, followed by dyspnoea and palpitations [1].

Pericardial cysts are located in the right heart border in 80%, less frequently in the left heart border (15%) and, rarely, in the anterior mediastinum [1].
